# Obesity and perioperative adverse events in patients undergoing complex revision surgery for the thoracolumbar spine

**DOI:** 10.1186/s12891-022-05505-4

**Published:** 2022-06-04

**Authors:** Takashi Hirase, Jeremiah F. Ling, Varan Haghshenas, Richard Fuld, David Dong, Darrell S. Hanson, B. Christoph Meyer, Rex A. W. Marco

**Affiliations:** 1 Department of Orthopedics and Sports Medicine, Houston Methodist Hospital, 6445 Main St Suite 2500, Houston, TX 77030 USA; 2 Texas A&M University Health Science Center College of Medicine, 8447 Riverside Pkwy, Bryan, TX 77807 USA

**Keywords:** Obesity, Revision thoracolumbar spine surgery, Low muscle mass, Complex, Predictor

## Abstract

**Background:**

There are no previous studies that evaluate the effect of obesity on patients undergoing complex revision thoracolumbar spine surgery. The primary objective was to determine the relationship between obesity and perioperative adverse events (AEs) with patients undergoing complex revision thoracolumbar spine surgery while controlling for psoas muscle index (PMI) as a confounding variable. The secondary objective was to determine the relationship between obesity and 30-day readmission rates, 30-day re-operation rates, rate of discharge to a facility, and post-operative length of stay (LOS).

**Methods:**

Between May 2016 and February 2020, a retrospective analysis of individuals undergoing complex revision surgery of the thoracolumbar spine was performed at a single institution. Obesity was defined as BMI ≥ 30.0 kg/m^2^. PMI < 500 mm^2^/m^2^ for males and < 412 mm^2^/m^2^ for females were used to define low muscle mass. A Spine Surgical Invasiveness Index (SSII) > 10 was used to define complex revision surgery. A multivariable logistic regression model was used to ascertain the effects of low muscle mass, obesity, age, and gender on the likelihood of the occurrence of any AE.

**Results:**

A total of 114 consecutive patients were included in the study. Fifty-four patients were in the obese cohort and 60 patients in the non-obese cohort. There was not a significant difference in perioperative outcomes of both the obese and non-obese patients. There were 22 obese patients (40.7%) and 33 non-obese patients (55.0%) that experienced any AE (*p* = 0.130).

Multivariable analysis demonstrated that individuals with low muscle mass had a significantly higher likelihood for an AE than individuals with normal or high muscle mass (OR: 7.53, 95% CI: 3.05-18.60). Obesity did not have a significant effect in predicting AEs.

**Conclusions:**

Obesity is not associated with perioperative AEs, 30-day readmission rates, 30-day re-operation rates, rate of discharge to a facility, or post-operative length of stay (LOS) among patients undergoing complex revision thoracolumbar spine surgery.

**Level of evidence:**

III

## Introduction

Currently, over 100 million adults living in the United States are considered obese and overweight [1]. The NIH utilizes body mass index (BMI) as a parameter of categorizing weight status, calculating BMI using body weight in kilograms dived by height in meters squared [2]. Obesity is a chronic disease recognized clinically as having a BMI > 30 in adults and has association with other comorbidities such as Type 2 Diabetes, cardiovascular disease and dyslipidemia [3]. The impact of obesity on spinal surgeries remains unclear, and complications related to obesity have been reported in some studies [4, 5]. Studies by Shamji et al. and Manson et al. found that obesity is associated with increased resources and perioperative transfusion requirements after elective thoracolumbar spine surgery; however, they reported no other associations between obesity and increased adverse events (AEs) [6, 7]. Another study by Yadla et al. investigating outcomes after primary elective thoracolumbar spine surgery found no associates between BMI and increased AEs [8]. Furthermore, a more recent study by Varshneya et al. reported that although obesity may be associated with overall health burdens outside of the perioperative environment, there is no significant relationship attributed between obesity and AEs after primary thoracolumbar deformity surgery [9].

Revision thoracolumbar spine surgeries have been associated with significantly higher perioperative AEs compared with primary surgeries [10–12]. An increase in surgical complexity has also been associated with higher perioperative AEs in spine surgery [13]. Therefore, identifying specific prognostic indicators for complex revision thoracolumbar spine surgery is important in order to stratify pre-operative risk for these patients. A recent study by Hirase et al. demonstrated that low muscle mass, defined by psoas muscle index (PMI) below 500 mm^2^/m^2^ for males and 412 mm^2^/m^2^ for females, is predictive of increased perioperative AEs among patients undergoing complex revision thoracolumbar spine surgery [14]. However, this study reported a significantly lower BMI among patients with low muscle mass, indicating the presence of a possible confounding factor for predicting perioperative AEs within this patient cohort. Our understanding is that there are no previous studies that evaluate the effect of obesity status on patients undergoing complex revision thoracolumbar spine surgery. Thus, the primary purpose of this study was to elucidate the relationship between obesity and perioperative AEs among patients undergoing complex revision thoracolumbar spine surgery while controlling for PMI as a confounding variable. The secondary objective was to determine the relationship between obesity and 30-day readmission rates, 30-day re-operation rates, rate of discharge to a facility, and post-operative length of stay (LOS).

## Materials and methods

The local institutional review board approved the study procedure on April 13, 2020. Due to the retrospective observational nature of this study, the informed consent was waived.

### Study population

Between May 2016 and February 2020, a retrospective analysis was performed at a single institution of patients receiving complex revision thoracolumbar spine surgery by three board certified fellowship-trained orthopaedic spine surgeons. The same inclusion and exclusion criteria for the same cohort of patients from our previous publication by Hirase et al. was applied for this study [14]. Any patients age 18 or above undergoing complex revision thoracolumbar spine surgery were included. Any history of prior surgical intervention of the same vertebral level was defined as revision surgery. A Spine Surgical Invasiveness Index (SSII) > 10 was used to define complex surgery [15]. Patients lacking a pre-operative CT or MRI of the lumbar spine obtained at our facility within 6 months of surgery, poor image quality, pre-operative MRI or CT acquired at any outside facilities, clinical evidence of L1 or L2 nerve root compression, a history of previous surgical treatment to or through the psoas muscle, and coronal deformity greater than 20 degrees were excluded as mentioned in our previous study [14].

### Data collection

Electronic medical records were utilized retrospectively to obtain demographic data including age, gender, BMI, American Anesthesiologists’ Society (ASA) class, comorbidities, indication for revision operation, baseline ambulation status, and presence of neurologic deficits. BMI ≥ 30.0 kg/m^2^ was used to define obesity. Intraoperative data was acquired which included estimated blood loss (EBL) and operative time defined as time of incision to post-operative dressing placement. SSII, a verified method of evaluation and comparison regarding spine surgery complexity was used, with a range of 0-48, which accounts for surgical approaches and the amount of decompressed, fused, and instrumented vertebral levels [15]. In regard to the included surgical population with revision surgeries, only the additional levels of fusion, instrumentation, or decompression were accounted for the SSII calculation as previously stated [14]. Each patients’ primary surgery SSII score was also obtained.

### Assessment of low muscle mass

PMI was used to analyze low muscle mass, which was calculated by measuring the total cross-sectional area (CSA) of the bilateral psoas muscles at the L3 vertebral body using the pre-operative T1 weighted MRI or CT normalized to body height [2] (mm^2^/m^2^). The total CSA was measured using OsiriX DICOM Viewer software (Version 11.0, Bernex, Switzerland) by manual outlining the bilateral psoas muscles at the first axial cut in the craniocaudal direction in which both transverse processes were visible at the L3 level as previously described [14, 16]. All of the images were obtained at a single institution with the same scanning protocols ensuring identical scanning thickness among all images analyzed. Three separate reviewers performed all measurements to improve interobserver reliability. Each measurement was acquired three times by all reviewers to improve intraobserver reliability. Intraclass correlation coefficient (ICC) was used to assess interobserver and intraobserver reliabilities, where an ICC above 0.90 signifies excellent agreement, between 0.75 and 0.90 signifies good agreement, between 0.5 and 0.75 signifies moderate agreement, and below 0.5 signifies poor agreement [17]. Each of the mean values obtained by the three reviewers was divided by the square of patient height to calculate the PMI as previously described [14]. To minimize the risk of bias, all reviewers were blinded to their respective measurements as well as to patient demographics and outcomes. Low muscle mass was defined as PMI < 500 mm^2^/m^2^ for males and < 412 mm^2^/m^2^ for females as previously defined [14].

### Outcome measures

Retrospective analysis of electronic medical records was used to review all perioperative outcomes. The primary outcome measures were perioperative AEs which included post-operative anemia that required transfusion, cardiac complication (cardiac arrest and myocardial infarction), sepsis, wound complication (wound dehiscence and deep wound infection), acute kidney injury (AKI), delirium, intra-operative dural tear, pneumonia, urinary tract infection (UTI), urinary retention, epidural hematoma, and deep vein thrombosis (DVT). The secondary outcome measures used were 30-day readmission rates, 30-day re-operation rates, in-hospital mortality rates, discharge disposition (home vs facility) and post-operative hospital length of stay (LOS). The number of days from surgery (or the last surgery if staged procedure) to discharge to either home or facility was used to defined postoperative LOS.

### Statistical analysis

SPSS statistical software (Version 25.0; SPSS, Inc., Chicago, IL) was utilized to perform data analysis. The Chi-Square or Fisher’s exact test was used to analyze categorical data and continuous data was analyzed using two-tailed student *t-*test. Continuous variables with non-normal distribution was analyzed using the Mann-Whitney U test. Statistical significance was set to *p*-value < 0.05. The odds ratio (OR) with 95% confidence interval (CI) was calculated for comparing perioperative outcomes. Post hoc power analysis with a two-tailed alpha of 0.05 was performed between obese and non-obese groups to evaluate the power of detecting differences between patients experiencing any perioperative. A multivariable logistic regression model was used to determine the effects of low muscle mass, obesity, age, and gender on the likelihood of the occurrence of any AE.

## Results

In total, there were 166 patients that met the inclusion criteria and 52 were removed based on the exclusion criteria. Final analysis included 114 patients (mean age 60.1 ± 15.4 years, 45 males, 69 females). The overall mean PMI was 495.0 ± 182.9 mm^2^/m^2^. Interobserver and intraobserver reliabilities were considered excellent with ICC of 0.908 (95% CI 0.862-0.944) and 0.962 (95% CI 0.928-0.975), respectively. Fifty-four patients were in the obese cohort and 60 patients in the non-obese cohort. The obese patients had a higher BMI and PMI compared to non-obese patients; otherwise, there were no significant differences in baseline demographics, comorbidities, presence of motor/sensory deficits, ambulatory status, indication for reoperation, or SSII between the two groups (Table 1). No significant difference between the perioperative outcomes was found among the obese and non-obese patients including adverse events (Table 2). There were 22 obese patients (40.7%) and 33 non-obese patients (55.0%) that experienced any AE (*p* = 0.130).

**Table 1:** Demographics and characteristics of the study population

Variable	Obese (***n*** = 54)	Non-obese (***n*** = 60)	***P***-value
BMI (kg/m^2^), mean ± SD (range)	36.2 ± 5.4 (30.0 – 51.7)	25.8 ± 3.0 (16.0 – 29.8)	< 0.001*
Male, n (%)	25 (46.3)	20 (33.3)	0.159
Age (y), mean ± SD (range)	61.4 ± 12.0 (20 – 77)	62.1 ± 13.3 (25 – 80)	0.768
ASA Class, mean ± SD (range)	2.78 ± 0.54 (2 – 4)	2.57 ± 0.65 (1 – 4)	0.060
PMI (mm^2^/m^2^), mean ± SD (range)	545.3 ± 174.4 (190.0 – 941.7)	449.6 ± 179.8 (152.2 – 940.4)	0.004*
Comorbidities/Pre-existing conditions, n (%)			
DM	11 (20.4)	10 (16.7)	0.611
Smokers	7 (13.0)	7 (11.7)	0.834
Cardiac (CHF, CAD, Atrial fibrillation)	11 (20.4)	14 (23.3)	0.704
CKD	7 (13.0)	9 (15.0)	0.757
COPD	19 (35.2)	15 (25.0)	0.234
CVA	1 (1.9)	1 (1.7)	0.936
PVD	2 (3.7)	1 (1.7)	0.497
Cancer	4 (7.4)	2 (3.3)	0.332
No pre-operative motor deficits, n (%)	41 (75.9)	39 (65.0)	0.204
No pre-operative sensory deficits, n (%)	35 (64.8)	30 (50.0)	0.110
Ambulatory status, n (%)			
Ambulate without assistive device	16 (29.6)	20 (33.3)	0.674
Ambulate with assistive device	30 (55.6)	23 (38.3)	0.066
Wheelchair bound	8 (14.8)	7 (11.7)	0.617
Not recorded	4 (7.4)	6 (10.0)	0.624
Indication for reoperation			
Spinal stenosis	22 (40.7)	29 (48.3)	0.418
Disc disease	13 (24.1)	16 (26.7)	0.749
Spondylolisthesis	14 (25.9)	14 (23.3)	0.749
Trauma	3 (5.6)	2 (3.3)	0.562
Deformity	25 (46.3)	27 (45.0)	0.889
Adjacent segment disease	22 (40.7)	24 (40.0)	0.936
Pseudarthrosis	27 (50.0)	30 (50.0)	1.000
HNP	3 (5.6)	3 (5.0)	0.897
Infection	2 (3.7)	1 (1.7)	0.497
SSII of primary surgery, mean ± SD (range)	11.9 ± 6.3 (3 – 32)	13.9 ± 5.9 (3 – 32)	0.067
SSII of revision surgery, mean ± SD (range)	25.6 ± 13.7 (11 – 48)	25.8 ± 11.3 (11 – 48)	0.943

*PMI* Psoas muscle index, *ASA* American Society of Anesthesiologists, *BMI* Body mass index, *DM* Diabetes mellitus, *CHF* Congestive heart failure, *CAD* Coronary artery disease, *CKD* Chronic kidney disease, *COPD* Chronic obstructive pulmonary disease, *CVA* Cerebrovascular accident, *PVD* Peripheral vascular disease, *HNP* Herniated nucleus pulposus, *SSII* Spine surgical invasiveness index [15]

*Statistically significant values

**Table 2:** Comparison of perioperative outcomes

Variable	Obese (***N*** = 54)	Non-obese (***n*** = 60)	***P*** Value	OR (95% CI)
Operative time (minutes), mean ± SD (range)	387.1 ± 224.3 (108-1160)	372.2 ± 172.7 (122-937)	0.693	N/A
EBL (mL), mean ± SD (range)	1235.5 ± 1792.4 (75-12,321)	856.2 ± 744.0 (50-4600)	0.152	N/A
EBL > 1 L, n (%)	22 (40.7)	19 (31.7)	0.490	1.29 (0.63-2.63)
Intraoperative pRBC transfusion units, mean ± SD (range)	2.2 ± 2.6 (0-15)	1.7 ± 1.6 (0-8)	0.238	N/A
Post-operative LOS (days), mean ± SD (range)	7.1 ± 5.6 (2-36)	6.3 ± 4.0 (1-19)	0.375	N/A
Discharge Disposition, n (%)				
Home	20 (37.0)	19 (31.7)	0.547	1.27 (0.59-2.76)
Facility	34 (63.0)	41 (68.3)	0.547	0.79 (0.36-1.71)
30-day Reoperation, n (%)	4 (7.4)	5 (8.3)	0.855	0.88 (0.22-3.46)
30-day Readmission, n (%)	4 (7.4)	6 (10.0)	0.626	0.72 (0.19-2.70)
In-hospital mortality, n (%)	0 (0.0)	1 (1.7)	0.539	0.36 (0.01-9.12)
Perioperative Adverse Events, n (%)				
Any adverse events	22 (40.7)	33 (55.0)	0.130	0.56 (0.27-1.18)
Post-op anemia requiring transfusion	8 (14.8)	18 (30.0)	0.058	0.41 (0.16-1.03)
Cardiac (cardiac arrest, MI)	1 (1.9)	2 (3.3)	0.487	0.55 (0.05-6.21)
Wound complication (dehiscence, infection)	4 (7.4)	7 (11.7)	0.763	0.61 (0.17-2.20)
Delirium	2 (3.7)	8 (13.3)	0.089	0.25 (0.05-1.23)
AKI	5 (9.3)	7 (11.7)	0.676	0.77 (0.23-2.59)
Pneumonia	3 (5.6)	6 (10.0)	0.386	0.53 (0.13-2.23)
UTI	2 (3.7)	9 (15.0)	0.059	0.21 (0.04-1.06)
Urinary retention	4 (7.4)	3 (5.0)	0.595	1.52 (0.32-7.12)
DVT	4 (7.4)	6 (10.0)	0.626	0.72 (0.19-2.70)
Dural tear/epidural hematoma/nerve injury	5 (9.3)	3 (5.0)	0.414	1.85 (0.42-8.12)

*OR* Odds ratio, *EBL* Estimated blood loss, *SD* Standard deviation, *CI* Confidence interval, *pRBC* Packed red blood cells, *LOS* Length of stay, *MI* Myocardial infarction, *AKI* Acute kidney injury, *UTI* Urinary tract infection, *DVT* Deep vein thrombosis

The multivariable logistic regression model was statistically significant, χ^2^(4) = 28.572, *p* < .0005. The model explained 29.6% (Nagelkerke *R*^2^) of the variance in any AE and correctly classified 72.8% of cases. The model revealed that individuals with low muscle mass had a significantly higher likelihood for an AE than individuals with a normal or high muscle mass (OR: 7.53, 95% CI: 3.05-18.60). Obesity, age, and gender did not have a significant effect in predicting AEs (Table 3). The post hoc power analysis between the obese and non-obese groups calculated a 95.2% power of detecting differences between patients experiencing any perioperative AEs.

**Table 3:** Multivariable logistic regression model to assess effects on adverse events (*n* = 114)

Variable	Reference Category for OR	Unadjusted OR (95% CI)	***P*** Value	Adjusted OR (95% CI)	***P*** Value
Obesity (BMI ≥ 30 kg/m^2^)	BMI < 30	0.61 (0.29-1.27)	0.187	1.04 (0.43-2.52)	0.929
Low muscle mass (male PMI < 500 mm^2^/m^2^, female PMI < 412 mm^2^/m^2^)	PMI ≥ 500 mm^2^/m^2^, female PMI > 412 mm^2^/m^2^)	7.46 (3.22-17.3)	< 0.001*	7.53 (3.05-18.6)	< 0.001*
Age (years)	N/A	1.04 (1.00-1.07)	0.064	1.03 (1.00-1.07)	0.065
Female gender	Male gender	1.18 (0.56-2.50)	0.672	0.94 (0.39-2.24)	0.882

*BMI* Body mass index, *PMI* Psoas muscle index, *OR* Odds ratio, *CI* Confidence interval

*Statistically significant values

## Discussion

In this study, the use of BMI as a predictor of perioperative AEs among patients undergoing complex thoracolumbar spine surgery was investigated. The multivariable logistic regression model confirmed that individuals with low muscle mass were at a significantly higher likelihood to experience AE compared to individuals with a normal or high muscle mass; however, there were no associations between BMI and perioperative AEs, 30-day readmission rates, 30-day re-operation rates, rate of discharge to a facility, or post-operative LOS among patients undergoing complex revision thoracolumbar spine surgery.

The relationship between obesity and perioperative AEs has been an area of debate over the past decade. Various studies have shown a significantly higher risk of AEs among obese patients after thoracolumbar surgery [15, 18–21]. The most recent study by Passias et al. determined that obese patients with a prior bariatric surgery had a significantly lower complication rate after thoracolumbar spine surgery compared with obese patients that did not undergo bariatric surgery [21]. Our findings contradict these series of studies as our analysis demonstrated no significant difference in post-operative AEs between obese and non-obese patients. There are likely multiple reasons that our study had different results. First, our study performed a multivariable analysis with low muscle mass, measured by PMI, which demonstrated a significantly higher association with post-operative AEs. None of the prior studies that investigated the association between obesity and AEs included low muscle mass as a potential confounding variable. Second, our patient cohort consisted solely of complex revision cases, which is a particularly unique population with high complication rates at baseline. From our study analysis, it may be speculated that amongst these complex patients, pre-operative low muscle mass, and the associated debility and frailty, is a much better predictor for post-operative complications compared with the risks associated with obesity. This can be speculated as obese patients within our cohort, had a significantly higher mean PMI compared to non-obese patients (*p* = 0.004, Table 1). Furthermore, studies have shown that although bariatric surgery is effective at lowering the BMI, this procedure may also induce low muscle mass post-operatively [22, 23]. Thus, with the known risks of low muscle mass shown within our study, it may be inadvisable to recommend bariatric surgery to improve BMI prior to undergoing complex revision thoracolumbar spine surgery.

A particularly important process among patients undergoing complex revision surgeries known to have high perioperative complications is preoperative risk stratification. Our previous study by Hirase et al. identified low muscle mass measured by PMI as a predictor of perioperative AEs among patients undergoing complex revision thoracolumbar spine surgery [14]. Our multivariable analysis within this study confirmed these findings and also demonstrated that obesity is not associated with perioperative AEs within this patient population. This combination of findings will specifically assist spine surgeons during pre-operative counseling and evaluation on two fronts. First, pre-operative optimization and overall conditioning to increase PMI among patients with low muscle mass may be beneficial to preventing post-operative AE. Second, obese patients may not benefit from aggressive weight loss prior to these surgeries, particularly, as studies have shown that improper weight loss methods may lead to low muscle mass [24, 25].

Our study has several limitations. Data accuracy is contingent on charting accuracy due to the retrospective nature of the study and may be susceptible to certain selection bias. Our results obtained from a single-center data may not be completely reflective of outcomes from other institutions due to variations in surgical technique or management. Our study also consisted of a relatively small patient cohort that may have led to underpowering to detect certain associations; however, our post hoc power analysis demonstrated that the absence of observed difference is unlikely due to lack of power. Furthermore, there may have been small discrepancies in inter-scan agreement between CT and MRI scans used to obtain the PMI; however, this is a previously established method with studies showing good inter-scan reliability with an ICC of 0.821 [14, 26]. Thus, to increase the power of the study, patients were included if they received either a CT or an MRI pre-operatively. Furthermore, although this study demonstrates that obesity is not a predictor of post-operative AEs within this surgical population, we cannot directly conclude that weight loss will not be beneficial in preventing post-operative AEs. Therefore, further studies that investigate the external validity of this study may be beneficial prior to application in practice.

In spite of the aforementioned limitations, to the best of our knowledge, this is the largest study examining the relationship between obesity and perioperative outcomes among patients receiving complex revision thoracolumbar spine surgery. This study will serve to assist spine surgeons with additional information during pre-operative counseling and evaluation regarding risks and benefits associated with low muscle mass and obesity. For patients undergoing complex revision thoracolumbar spine surgery, our findings may suggest that the benefits of weight loss among obese patients may not outweigh the risk of inducing low muscle mass prior to the operation.

## Conclusions

Obesity is not associated with perioperative AEs, 30-day readmission rates, 30-day re-operation rates, rate of discharge to a facility, or post-operative length of stay (LOS) among patients undergoing complex revision thoracolumbar spine surgery.

## Data Availability

The datasets generated and/or analyzed during the current study are not publicly available due to departmental patient privacy policy. Due to the complex and unique nature of each patient presented in this work, patients remain vulnerable to identification despite the removal of patient identifying information. However, the database will be available from the corresponding author on reasonable request.

## Abbreviations

AEs: Adverse events
PMI: Psoas muscle index
LOS: Length of stay
BMI: Body mass index
SSII: Spine surgical invasiveness index
ASA: American Anesthesiologists’ Society
EMR: Electronic medical records
EBL: Estimated blood loss
CSA: Cross-sectional area
ICC: Intraclass correlation coefficient
AKI: Acute kidney injury
UTI: Urinary tract infection
DVT: Deep vein thrombosis
OR: Odds ratio
CI: Confidence interval
